# Hereditary pancreatic cancer

**DOI:** 10.1007/s10147-021-02015-6

**Published:** 2021-09-02

**Authors:** Kodai Abe, Minoru Kitago, Yuko Kitagawa, Akira Hirasawa

**Affiliations:** 1 Department of Surgery, Keio University School of Medicine, Tokyo, Japan; 2 Department of Clinical Genomic Medicine, Graduate School of Medicine, Dentistry and Pharmaceutical Sciences, Okayama University, 2-5-1 Shikata-cho, Kita-ku, Okayama, 700-8558 Japan

**Keywords:** Cancer predisposition gene, Familial pancreatic cancer, Hereditary pancreatic cancer, Multigene panel testing, Surveillance

## Abstract

Pancreatic cancer is associated with both family and hereditary cancer syndromes. Multigene panel testing for pancreatic cancer detected the germline variants *BRCA1/2*, *PALB2*, *ATM*, *TP53*, *MLH1*, *STK11/LKB1*, *APC*, *CDKN2A*, and *SPINK1/PRSS1* as high-risk genes. A latest genome-wide association study revealed the common, but low-risk germline variants in pancreatic cancer patients. Active pancreatic surveillance using magnetic resonance imaging and endoscopic ultrasound is recommended for high-risk individuals who have a family history of pancreatic cancer or harbor these germline pathogenic variants to improve the detection rate and prognosis of pancreatic cancer. Since poly-ADP-ribose polymerase (PARP) inhibitor has been shown to be effective in improving the prognosis of *BRCA*-positive pancreatic cancer as well as hereditary breast and ovarian cancer syndrome, PARP inhibitor therapy is currently being applied as precision medicine to pancreatic cancer patients harboring the *BRCA1/2* germline variant. This review highlights the importance of surveillance for germline pathogenic variants in pancreatic cancer and is expected to lead to improvements in the diagnosis and prevention of pancreatic cancer as well as facilitate the development of effective therapeutic strategies and precision medicine.

## Introduction

**Fig. 1:**
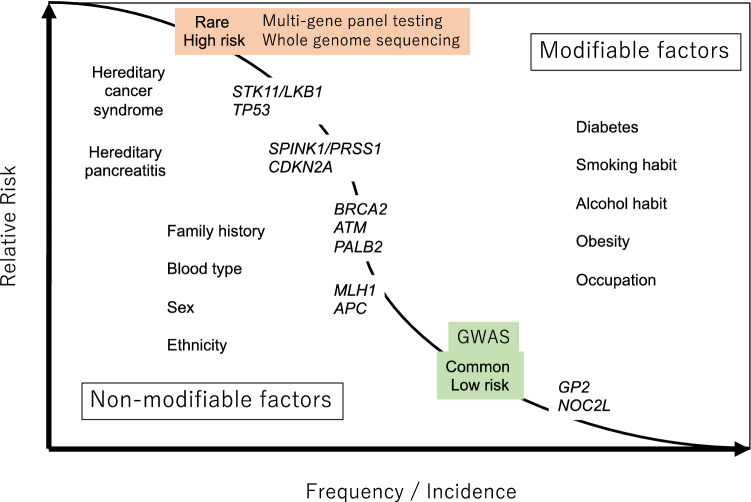
Relative risk and incidence of risk factors for pancreatic cancer. Pancreatic cancer has multiple mixed risk factors, including modifiable and nonmodifiable factors. These risk factors correlate with germline pathogenic variants. Very rare, but extremely high-risk germline variants (*STK11/LKB1*, *TP53*, and *PRSS1/SPINK1*) are usually detected using family linkage analysis, and rare and high-risk germline variants (*BRCA1/2*, *ATM*, *PALB2*, *CDKN2A*, *MLH1*, and *APC*) are detected using multigene panel testing. Common low-risk germline variants (*GP2*, and *NOC2L*) are detected using genome-wide associated studies

Pancreatic cancer is a multifactorial genetic disease. There are known germline pathogenic variants associated with hereditary pancreatic cancer, which is closely related to other hereditary tumor syndromes. Several high-risk factors have been identified, including family history of pancreatic cancer [1]. These risk factors are divided into modifiable, including smoking, daily alcohol consumption, chronic pancreatitis, diet, and obesity; and nonmodifiable, including age, gender, ethnicity, ABO blood group, diabetes, and family history (Fig. 1) [2–4]. Familial pancreatic cancer is defined as having two or more first-degree relatives (FDRs) with pancreatic cancer and accounts for approximately 3%–8.4% of all cases of this disease [5, 6]. Genome sequencing analysis is used to investigate the germline variants of pancreatic cancers. In addition, genome-wide association analysis has identified common single nucleotide variants (SNVs) that are more frequently detected in pancreatic cancer.

Germline variant data can support early detection, prevention, and personalized treatment of pancreatic cancer. Here, we highlight the most recently identified germline variants in pancreatic cancer, current management strategies, and future perspectives for improving prognosis.

## Hereditary pancreatic cancer related genes

Genes associated with hereditary cancer plays a key role in the development of pancreatic cancer [7–10]. The relationship between various hereditary cancer syndromes and pancreatic diseases is shown in Table 1. These pancreatic diseases can serve as prognostic determinants for each hereditary cancer syndrome.

**Table 1:** Relationship between germline variants and pancreatic diseases

Gene	Hereditary cancer syndrome	Pancreatic disease(s)	Relative risk for pancreatic cancer	Cumulative incidence of pancreatic cancer (%)	Phenotype	References
*BRCA1*	Hereditary breast and ovarian cancer syndrome	Pancreatic cancer	2.26–3	1–3% (life-time risk)	Breast, ovarian, and prostate cancer	[13, 14]
*BRCA2*			3.5–10	2–7% (life-time risk)		
*PALB2*	–	Pancreatic cancer Intraductal papillary mucinous neoplasm	2.37	2–3% (up to 80 years of age)	Breast, ovarian, and prostate cancer	[15, 17]
*ATM*	–	Pancreatic cancer	2.41	N.D	Breast, ovarian, and prostate cancer	[18]
*NBN*	–	Pancreatic cancer	N.D	N.D	Breast, ovarian, and prostate cancer	[15]
*TP53*	–	Pancreatic cancer	7.3	N.D	Adrenocortical carcinoma	[21]
					Breast cancer	
					Central nervous system tumor	
					Osteosarcoma	
					Soft tissue sarcoma	
*MLH1*	Lynch syndrome	Pancreatic cancer	7.8	6.2% (up to 75 years of age)	Colorectal, gastric, and endometrial cancer	[24]
*MSH2*			0.6	0.5% (up to 75 years of age)		
*MSH6*			1.8	1.4% (up to 75 years of age)		
*PMS2*			N.D	N.D		
*STK11/LKB1*	Peutz-Jeghers syndrome	Pancreatic cyst	76.2	25.6% (up to 70 years of age)	Lip pigmentation Gastrointestinal polyposis	[25, 26]
		Intraductal papillary mucinous neoplasm Pancreatic cancer			Colorectal, gastric, ovarian, breast, and cervical cancer	
*APC*	Familial adenomatous polyposis	Pancreatic cyst	4.5	N.D	Gastrointestinal polyposis	[27, 28]
		Intraductal papillary mucinous neoplasm Pancreatic cancer			Colorectal and duodenal cancer	
					Desmoid tumor	
*CDKN2A*	Familial atypical multiple	Pancreatic cancer	13–39	17% (up to 75 years of age)	Malignant melanoma	[30]
	mole melanoma syndrome				Lung cancer	
*SMAD4/BMPR1A*	Juvenile polyposis syndrome	Pancreatic cancer	N.D	N.D	Gastrointestinal polyposis	[30, 31]
*PRSS1/SPINK1*	Hereditary pancreatitis	Acute pancreatitis Chronic pancreatitis	53	40% (up to 70 years of age)	Pancreatitis	[32]

### BRCA1/2

*BRCA1* and *BRCA2* are associated with hereditary breast and ovarian cancer syndrome (HBOC). HBOC follows an autosomal dominant pattern of inheritance and is characterized by early-onset breast and ovarian cancers. *BRCA1/2* are DNA damage repair genes whose monoallelic or biallelic variants can cause HBOC or Fanconi anemia, respectively [11, 12]. Pathogenic *BRCA* carriers should undergo surveillance for breast, ovarian, prostate, and pancreatic cancer as well as melanoma. The incidence of germline *BRCA1/2* pathogenic variants in pancreatic cancer is 5%–9% [13]. Moreover, the relative risk (RR) and cumulative incidence of pancreatic cancer for germline *BRCA2* pathogenic variants are 3.5–10 and 2%–7%, and for germline *BRCA1* pathogenic variants, 2.26 and 1%–3%, respectively [14].

### PALB2

*PALB2* (partner and localizer of BRCA2) is also involved in DNA damage repair and Fanconi anemia, which is strongly associated with breast, ovarian, and pancreatic cancer [15, 16]. *PALB2* pathogenic germline variants are associated with a 7.18, 2.91, and 2.37 RR of developing female breast, ovarian, and pancreatic cancer, respectively, and their estimated incidence up to age 80 years is 53%, 5%, and 2%–3%, respectively [17]. The latest American College of Medical Genetics and Genomics guidelines recommend that pancreatic cancer surveillance should be considered for germline *PALB2* pathogenic variant [18].

### ATM

*ATM*, whose biallelic variant can cause Ataxia-telangiectasia, plays a role similar to that of *BRCA1/2* in DNA damage repair [19]. The *ATM* pathogenic germline variant is strongly associated with breast, ovarian, prostate, and pancreatic cancer. The RR for pancreatic cancer is 2.41 in individuals with the *ATM* germline pathogenic variant [20].

### NBN

*NBN* is a DNA damage response gene, whose biallelic variant can cause severe microcephaly, infertility, and cancer. *NBN* pathogenic variants are mostly reported in breast cancer, but recent reports link them to pancreatic cancer as well. However, the cumulative incidence or RR has not been reported [16, 21].

### TP53

The *TP53* pathogenic variant is known to cause Li–Fraumeni syndrome, which is associated with a high risk of developing adrenocortical carcinomas, hematopoietic malignancies, breast cancer, central nervous system tumors, osteosarcomas, and soft-tissue sarcomas (https://www.ncbi.nlm.nih.gov/books/NBK1311/). The *TP53* germline variant is associated with pancreatic cancer with RR of 7.3 [22].

### Mismatch repair (MMR) genes (*MLH1, MSH2, MSH6, PMS2*)

Defective MMR genes, such as *MLH1*, *MSH2*, *MSH6*, and *PMS2*, display microsatellite instability and are the susceptibility genes of Lynch syndrome [23], a hereditary syndrome associated with an increased predisposition to colorectal, gastric, and endometrial cancers. The accumulated risk of pancreatic cancer in Lynch syndrome is approximately 3.7%, which is slightly lower than that in HBOC [24]. The cumulative incidence of pancreatic cancer in MLH1 carriers up to age 75 years is higher (RR 7.8) than that observed for other MMR genes [25].

### STK11/LKB1

The *STK11/LKB1* germline variant accounts for approximately 90% of Peutz–Jeghers syndrome (PJS), which is a hereditary polyposis syndrome with an autosomal dominant pattern of inheritance. PJS patients have increased risk of gastrointestinal and nongastrointestinal cancers and extremely high risk of developing early-onset pancreatic cancer. The cumulative risk for pancreatic cancer is 25.6% up to the age of 70 years (hazard ratio 76.2), and the mean age of onset is 40.8 (SD 16.2) years [26]. Further, lobular endocervical glandular hyperplasia and peritoneal pigmentation are caused by the *STK11* germline variant [27].

### APC

*APC* is a well-known susceptibility gene for familial adenomatous polyposis (FAP) [28], a hereditary polyposis syndrome with an autosomal dominant pattern of inheritance. Polyps occur mainly in the colon, but gastric, duodenum, and small bowel polyps are also frequent. The RR for pancreatic cancer is 4.5 times of the general population [29].

### Cyclin-dependent kinase inhibitor 2A (*CDKN2A*)

*CDKN2A* is a major susceptibility gene for familial atypical multiple mole melanoma syndrome (FAMMM), and FAMMM kindreds are at high risk of developing pancreatic cancer. Individuals with *CDKN2A* variants have a 70% life-time risk of developing melanoma and a 17% risk of developing pancreatic cancer up to the age of 70 years [30]. The RR of developing pancreatic cancer was reported as 13–39 for individuals with mutations in *CDKN2A* [31, 32]; however, there are no specific screening guidelines for FAMMM.

### *SMAD4* and *BMPR1A*

The *SMAD4* germline variant is the most frequently recognized in juvenile polyposis syndrome (JPS), along with the *BMPR1A* germline variant [33, 34]. Although the RR of pancreatic cancer in JPS is not as high as in other hereditary polyposis syndromes, careful management for preventing pancreatic cancer may be needed.

### *PRSS1* and *SPINK1*

PRSS1 is encoded by the cationic trypsinogen gene, and *SPINK1* encodes the serine protease inhibitor Kazal 1. Hereditary pancreatitis is a rare disease that can progress to chronic pancreatitis. In Japan, the cumulative incidence of pancreatic cancer for hereditary pancreatitis with the *PRSS1* and *SPINK1* variants was 40% up to the age of 70 years, and the RR for pancreatic cancer in hereditary pancreatitis was 53 [35].

### CPA1 and CPB1

*CPA1* (encoding carboxypeptidase A1) and *CPB1* (encoding carboxypeptidase B1) can cause pancreatic acinar cell endoplasmic reticulum (ER) stress and these germline variants may lead to impaired secretion of its protein product and induce ER stress. These variants are more common in patients with pancreatic cancer (approximately 1% of individuals with pancreatic cancer when compared with < 0.001% in population controls), suggesting pancreatic acinar cell stress as a potential mechanism of pancreatic cancer susceptibility [36]

## Multigene panel testing

Multigene panel testing is an effective tool for identifying germline pathogenic variants of pancreatic cancer. First, a National Familial Pancreas Tumor Registry (NFPTR; http://pathology.jhu.edu/pancreas/nfptr/index.php) analysis of the germline DNA of 638 familial pancreatic cancer patients from 593 kindreds revealed that germline pathogenic variants of *ATM*, *BRCA2*, *CDKN2A*, and *PALB2* elevated the risk of pancreatic cancer, and that variants of *BUB1B*, *CPA1*, *FANCC*, and *FANCG* are detected more frequently in familial than in sporadic pancreatic cancer [37]. *BRCA2* and *ATM* germline pathogenic variants are significantly more prevalent in familial pancreatic cancer patients [38]. Furthermore, using linkage analysis, the palladin gene was reported as a new germline pathogenic variant in 2006 [39].

New germline pathogenic variants were described in Japanese familial pancreatic cancer patients [40]. In whole-exome sequencing of germline DNA from whole blood of 81 patients, *ATM* and *BRCA2* were recognized in 7 (8.6%) and 3 (3.7%) patients, respectively. Furthermore, *ASXL1* or *DNMT3A*, which are tumor suppressor genes, were also identified as germline variants of familial pancreatic cancer. Interestingly, new germline susceptibility genes, *FAT1* (*n* = 4) and *FAT4* (*n* = 3), which encode the large transmembrane proteins protocadherins, were discovered in seven patients [40]. Therefore, familial pancreatic cancer has a wide range of germline variants instead of a predominant gene such as *BRCA1/2* in HBOC or *APC* in FAP. Thus, multigene panel testing for pancreatic cancer patients may be able to provide substantial information about high-risk pathogenic germline variants common to previously known hereditary cancer syndromes.

## Low-risk germline variants and environmental factors

Genome-wide association studies (GWASs) have led to the discovery of common and low-risk germline variants in pancreatic cancer patients. A meta-analysis of GWASs conducted in Europe and the United States reported that an SNV of *NOC2L* (1p36.33, rs13303010) was significantly associated with pancreatic cancer [41]. A GWAS conducted in East Asia, including Japan and China, reported that an SNV of *GP2* (16p12.3, rs78193826), which is common in patients with pancreatic cancer is also strongly involved in KRAS activity [42]. Hence, it is important to recognize the environmental risk factors for pancreatic cancer because they can be modulated/targeted to prevent malignant progression.

## Registries for familial pancreatic cancer

Familial pancreatic cancer is as important as hereditary pancreatic cancer because a family history of pancreatic cancer places an individual at an extremely high risk of pancreatic cancer development. Familial pancreatic cancer was first reported in 1976 in the United States [43], leading to the suggestion of a genetic component. In 1994, the NFPTR was established by researchers in Johns Hopkins University, and prospective registration of familial pancreatic cancer was initiated (Table 2) [44, 45]. Subsequently, the European Registry of Hereditary Pancreatitis and Familial Pancreatic Cancer (EUROPAC, https://www.lctu.org.uk/LCTU_NET/frontend/Default.aspx?Data=W1tiRzlqWVd4bF1dW09RPT1d) was established in 1997 in the United Kingdom, and the German National Case Collection for Familial Pancreatic Carcinoma (FaPaCa, http://www.fapaca.de/) was introduced in 1999. The PanGen-Fam registry was established in Spain in 2009 to identify families with high PDAC risk [46]. In Japan, the registration system was initiated in 2014 as the Japan Familial Pancreatic Cancer Registry (JFPCR, http://jfpcr.com/) [47].

**Table 2:** Familial pancreatic cancer registry worldwide

Registration	Year of establishment	Country	References
National Familial Pancreas Tumor Registry	1994	United States	[19, 20]
The European Registry of Hereditary Pancreatitis and Familial Pancreatic Cancer	1997	United Kingdom	[21]
The German National Case Collection for Familial Pancreatic Carcinoma	1999	Germany	[22]
PanGen-Fam Registry	2009	Spain	[23]
Japan Familial Pancreatic Cancer Registry	2014	Japan	[18]

The first epidemiological study of NFPTR revealed that the risk in familial pancreatic cancer kindreds was elevated in individuals with three (odds ratio 32.0), two (odds ratio 6.4), or one (odds ratio 4.6) FDR(s) with pancreatic cancer [48, 49]. Next, the large EUROPAC cohort study in 2006 demonstrated that patients with familial pancreatic cancer experienced early onset of pancreatic cancer and were likely to be deceased younger than patients with sporadic pancreatic cancer, which was expressed as anticipation [50]. Third, FaPaCa showed that 74% of familial pancreatic cancer has an autosomal dominant inheritance pattern, and familial pancreatic cancer is frequently concomitant with breast cancer [51]. The analysis of the PanGen-Fam registry indicated that family history of cancer and diabetes increased the risk of pancreatic cancer development [52]. Thus, since the risk of developing pancreatic cancer is extremely high in cases with a strong family history, familial and hereditary pancreatic cancer cases should be approached aggressively.

## Surveillance for pathogenic germline variant carriers

Pancreatic cancer has one of the highest mortality rates among all types of malignant solid tumor. The 5-year overall survival is 9%–13% [53], and only approximately 25% of tumors are resectable [54]. Most patients display no symptoms and undergo rapid progression [55]. Thus, early diagnostic management and intervention is a must for improving outcomes.

The international cancer of the pancreas screening (CAPS) consortium proposed a concise strategy for pancreatic surveillance of high-risk individuals to improve the rate of detection at an early stage [56]. According to CAPS, the best imaging techniques for routine follow-ups are endoscopic ultrasound (EUS) and magnetic resonance imaging or cholangiopancreatography (MRI/MRCP) because of their higher sensitivity and specificity than that of computed tomography [57, 58]. It is suggested that these screenings should be initiated at the age of 30–35 years or 10 years younger than the earliest exocrine pancreatic cancer diagnosis in the family, especially for patients with PJS [6, 59], but the best timing to start screening of the pancreas for high-risk individuals remains to be established (Table 3) [60].

**Table 3:** High-risk individuals for pancreatic cancer

Subject categories	Start screening [59, 62] (age in years)
Three or more first- and/or second- degree relatives with pancreatic cancer	50
Two or more first-degree relatives with pancreatic cancer	50
Pathogenic germline variant carrier and a family history of pancreatic cancer (*ATM, BRCA1, BRCA2, MLH1, MSH2, MSH6, EPCAM, PALB2, TP53*)	50
Individuals with pathogenic germline variant in *STK11*	30–35
Individuals with pathogenic germline variant in *CDKN2A*	40

This surveillance is also recommended for individuals with three or more blood relatives with pancreatic cancer, and at least one of those affected FDR carrying the *BRCA2* or *PALB2* germline pathogenic variant. Surveillance should be considered for individuals with two affected blood relatives with pancreatic cancer or with at least one FDR [56]. In a 2018 study, during the median 5.6-year follow-up, 14 (4.0%) and 10 (2.8%) patients of the 354 high-risk individuals developed pancreatic cancer and high-grade dysplasia, including IPMN, respectively. The resection rate of the pancreatic cancers was 71.4%, and the 5-year overall survival rate was 60%, which is much better than the reported surveillance, epidemiology, and end results in the United States [61]. As this aggressive surveillance has a favorable prognosis, the latest NCCN guidelines also recommend EUS, MRI, and MRCP twice a year for high-risk individuals [62]. It is important to explore other diagnostic modalities including biomarkers to improve outcomes in the near future.

## Drug sensitivity

### Poly-ADP-ribose polymerase (PARP) inhibitors

PARP inhibitors play a key role in the induction of double-stranded breaks after the collapse of the DNA replication process. The efficacy of PARP inhibitors against advanced breast, ovarian, pancreatic, and prostate cancer with a *BRCA1/2* pathogenic variant was recognized in 2015 [63]. When compared with placebos, PARP inhibitors resulted in longer progression-free survival for *BRCA*-positive metastatic pancreatic cancer following platinum-based chemotherapy [63, 64]. Hence, PARP inhibitors are used as maintenance therapy and *BRCA* genetic testing for pancreatic cancer is a companion diagnosis.

### Immune checkpoint inhibitors

Pembrolizumab, an immune checkpoint inhibitor, has a critical impact and benefits the survival of patients with germline pathogenic variants of MMR genes [65]. Although only a small proportion (approximately 1%) of pancreatic cancer patients have these MMR genes, it is important to assess the potential efficacy of pembrolizumab in hereditary pancreatic cancer [66].

### Platinum

Patients with strong family history of breast, ovarian, and pancreatic cancer have better prognoses than those without such history and are sensitive to platinum-based chemotherapy [67]. However, a prospective Phase II study from Japan revealed that gemcitabine plus oxaliplatin did not produce a favorable outcome in patients with metastatic pancreatic cancer with a family history of breast, ovarian, and pancreatic cancer [68]. *BRCA* germline pathogenic variants are prognostic and therapeutic biomarkers in breast and ovarian cancer, and platinum-based chemotherapy is a standard treatment for these cancers [68]. As the overall survival for stage-3 or -4 *BRCA*-mutated pancreatic cancer was improved by platinum-based treatment [69], HBOC pancreatic cancer patients are good candidates for platinum-based therapy.

## Future perspectives for hereditary pancreatic cancer

There is no standard conceptualization about the disclosure of germline pathogenic variants. As mentioned earlier, it is possible to detect various types of deleterious variants in the DNA of familial pancreatic cancer patients. These need to be carefully analyzed to decide how to proceed with disease management [70]. Deleterious variants have also been reported in ~ 5%–10% of patients with apparently sporadic pancreatic cancer [31, 71]. Because of these papers, recent NCCN guidelines recommend offering genetic testing for patients with newly diagnosed pancreatic ductal adenocarcinoma [62] In addition, optimal surveillance is needed similar to that for HBOC syndrome, because pancreatic lesions are not usually found in routine medical check-ups [72].

It is difficult to predict germline pathogenic variants from a family history of pancreatic cancer. For example, when 306 pancreatic cancer patients were screened for *BRCA* genetic analysis, 14 (4.6%) were positive, of which only two had family history of pancreatic cancer, indicating that the absence of family history of pancreatic cancer does not mean that *BRCA* testing should not be performed [73]. *BRCA2*, *ATM*, and *BRCA1* were detected, in that order, from blood samples of 1 009 pancreatic cancer patients. However, the prediction of germline variants from clinical information such as sex, past medical history, and family history—easy for breast cancer—is extremely difficult for pancreatic cancer [74]. Furthermore, a recent study has revealed that harboring a pathogenic, or likely pathogenic variant is associated with a greater incidence of pancreatic cancer than family history alone (without the presence of an associated germline variant) [75]. Therefore, while hereditary and familial pancreatic cancer overlap in some cases, they must be considered as similar but different domains.

Finally, preventive total pancreatectomy for individuals with *BRCA1/2* germline pathogenic variants has not been allowed, as it can result in severe and potentially fatal complications, including insulin-dependent diabetes, pancreatic enzyme insufficiency, and blood glucose instability. Conversely, in the recent years, medical developments have led to better control of total pancreatectomy treatment and a trend toward low complication and mortality rates. Therefore, in Europe, it has been suggested that the decision program of prophylactic total pancreatectomy could be useful in high-risk individuals of pancreatic cancers such as hereditary pancreatitis and IPMN with *STK11/LKB1* or *CDKN2A* pathogenic variants [76].

## Conclusion

A deeper understanding of hereditary pancreatic cancer is crucial in improving strategies for detecting pancreatic cancer more frequently at an early stage and preventing malignant progression. Multigene panel testing or whole-genome sequencing should be performed for identifying germline pathogenic variants. Continuously updating the current knowledge base of hereditary pancreatic cancer syndromes will aid in the development of potential diagnostic and prognostic measures, resulting in more favorable outcomes.
